# Production of Lignin-Derived Functional Material for Efficient Electromagnetic Wave Absorption with an Ultralow Filler Ratio

**DOI:** 10.3390/polym16020201

**Published:** 2024-01-10

**Authors:** Yuebin Xi, Xingxiang Ji, Fangong Kong, Tianjin Li, Binpeng Zhang

**Affiliations:** 1 Key Laboratory of Pulp and Paper Science and Technology of Ministry of Education, State Key Laboratory of Biobased Material and Green Papermaking, Qilu University of Technology (Shandong Academy of Sciences), Jinan 250353, China; 2 Energy Institute, Qilu University of Technology (Shandong Academy of Sciences), Jinan 250353, China; 3 School of Chemistry and Chemical Engineering, Henan University of Technology, Zhengzhou 450001, China

**Keywords:** lignin, functional material, three-dimensional porous, carbon material, electromagnetic wave absorption

## Abstract

Industrial lignin, a by-product of pulping for papermaking fibers or of second-generation ethanol production, is primarily served as a low-grade combustible energy source. The fabrication of high-value-added functional materials with industrial lignin is still a challenge. Herein, a three-dimensional hierarchical lignin-derived porous carbon (HLPC) was prepared with lignosulfonate as the carbon source and MgCO_3_ as the template. The uniform mixing of precursor and template agent resulted in the construction of a three-dimensional hierarchical porous structure. HLPC presented excellent electromagnetic wave (EMW) absorption performance. With a low filler content of 7 wt%, HLPC showed a minimum reflection loss (*RL*) value of −41.8 dB (1.7 mm, 13.8 GHz), and a maximum effective absorption bandwidth (EAB) of 4.53 GHz (1.6 mm). In addition, the enhancement mechanism of HLPC for EMW absorption was also explored through comparing the morphology and electromagnetic parameters of lignin-derived carbon (LC) and lignin-derived porous carbon (LPC). The three-dimensional hierarchical porous structure endowed the carbon with a high pore volume, resulting in an abundant gas–solid interface between air and carbon for interfacial polarization. This structure also provided conductive networks for conduction loss. This work offers a strategy to synthesize biomass-based carbon for high-performance EMW absorption.

## 1. Introduction

Due to the widespread use of wireless communication techniques based on high-frequency electromagnetic waves (EMWs), electromagnetic pollution has become a rising concern. High-frequency EMWs are not only a threat against human health, but also cause electromagnetic interference for precision electronics [1,2,3]. To mitigate undesired EMWs, EMW-absorbing materials are always used by converting ambient EMWs into Joule heat [4,5]. In recent years, extensive studies have been carried out to develop EMW-absorbing materials with excellent performance, including strong attenuation, broad absorption bandwidth, thin thickness and light weight [6]. In addition, the environmental adaptability of the absorber is also important due to complex environments in practical applications [7,8].

Currently, many kinds of absorbers have been fabricated, such as magnetic materials [9,10], ceramic materials [11,12], carbon-based materials [13,14], conductive polymers [15] and so on. Among the absorbers, carbon-based materials have drawn extensive attention because of their inherent features such as low density, high electrical conductivity and good stability. These features make them potential candidates for lightweight and stable EMW-absorbing materials [16]. Carbonaceous absorbers are regarded as dielectric absorbing materials according to the loss mechanism. The absorption performance for EMWs is related to the impedance-matching characteristic and attenuation capacity of carbonaceous materials. After the incident EMW reaches the surface of carbonaceous absorbers, a portion of the EMW will immediately reflect due to the impedance mismatch. The remaining EMW can reflect and refract multiple times inside the material, and is then attenuated via conduction loss and dielectric polarization loss [17,18,19].

Biomass-derived carbon presents superiority because of rich sources and environmental friendliness compared with other carbonaceous materials, which makes them more suitable for practical application [20,21,22]. Therefore, many biomasses, such as lignocellulose [23], chicken feather fibers [24], rice [25], eggs [26] and so on, have been used to prepare carbonaceous materials for EMW absorption. Luo et al. prepared hierarchically porous carbon derived from natural Porphyra, and the minimum reflection loss reached −57.75 dB at 9.68 GHz (3.3 mm) with a filling ratio of 30 wt% [27]. Li et al. fabricated biomass-derived carbon composites with a loofah sponge, and the composite presented a minimum reflection loss of −43.8 dB at 8.3 GHz (3 mm) with a filling ratio of 30 wt% [28]. The biomass-derived carbon exhibited excellent reflection loss for EMWs, but the lightweight and thin thickness features were difficult to achieve simultaneously.

For carbon-based materials, high intrinsic conductivity endows them with excellent conductivity loss [29,30]. However, the high electrical conductivity also causes high complex permittivity, which results in impedance mismatch [31,32]. Thus, a lot of incident EMW can be reflected, leading to poor EMW attenuation. It is an effective strategy to improve the EMW absorption performance of carbon-based absorbers by introducing the magnetic loss mechanism (natural ferromagnetic resonance and eddy current effect). Magnetic metal (Fe, Co, Ni, etc.) and ferrite (Fe_3_O_4_, etc.) are always combined with carbonaceous materials for tuning the impedance matching characteristics and attenuation ability [33]. But magnetic materials present a high filling ration and poor stability compared with carbonaceous materials. The obtained composites had difficulties in meeting the lightweight and stability needs in practice.

In addition to the composite strategy, the rational control of microstructures can optimize impedance matching and the EMW loss capability of carbonaceous materials [34]. According to the Maxwell–Garnett theory, the porous structure of absorbers can reduce the effective permittivity because of the increase in free spaces, and then increase the EMWs entering the material. In addition, the porosity creates numerous solid–air interfaces, resulting in an increase in interfacial polarization. The porosity also causes multiple reflections and scattering of EMWs, expanding the attenuation pathways [35,36]. Based on the above analysis, pore volume plays a crucial role in EMW absorption for carbon-based materials. However, there are few studies on the biomass-derived porous carbon with high pore volume due to the limited fabrication method.

Lignin, as the world’s second-most abundant biopolymer after cellulose, is a type of environmentally friendly biomass resource. Industrial lignin is mainly derived from the by-product of pulp and the paper industry, including lignosulfonate produced in the sulfite pulping process and Kraft lignin produced in the Kraft pulping process. In addition, with the rapid growth of the cellulosic bioethanol industry, enzymatic hydrolysis lignin accounts for an increasing proportion of industrial lignin [37]. Currently, more than 50 million tons of industrial lignin is produced annually, but a large amount of industrial lignin is discarded into the environment as a castoff or burned for energy, resulting in resource waste and environmental pollution.

Lignin is an aromatic polymer that mainly includes three principal monomers (guaiacyl, p-hydroxyphenyl and syringyl). The carbon content of lignin can reach up to 60%. Thus, lignin is an ideal carbon precursor [38]. The different processes result in different types of industrial lignin. The properties of these lignins can vary depending on the manufacturing processes. Lignosulfonates are water soluble due to the introduction of sulfonic groups on the lignin structure during the sulfite pulping process. Lignosulfonates can achieve uniform mixing with templates or activators in an aqueous solution compared with Kraft lignin and enzymatic hydrolysis lignin. Therefore, lignosulfonates present a better flexibility and designability as the carbon precursor. Thus, lignosulfonates have great potential for the preparation of high value-added carbon-based function materials.

Herein, we prepared a three-dimensional hierarchical lignin-derived porous carbon (HLPC) with lignosulfonate as the carbon source and MgCO_3_ as the template. The uniform mixing of the precursor and template agent was achieved through the adsorption and coprecipitation strategy. After carbonization, the three-dimensional hierarchical porous structure was constructed, and this unique structure endowed HLPC with an ultrahigh pore volume. With a low filler content of 7 wt%, HLPC showed a minimum reflection loss (*RL*) value of −41.8 dB (1.7 mm, 13.8 GHz), and a maximum effective absorption bandwidth (EAB) of 4.53 GHz (1.6 mm). In addition, the enhancement mechanism of HLPC for EMW absorption was also explored through comparing the morphology and electromagnetic parameters of lignin-derived carbon (LC) and lignin-derived porous carbon (LPC). This work indicated the EMW absorption potential of single carbonaceous material and manifested the superiorities of HLPC in practical EMW absorption applications.

## 2. Materials and Methods

### 2.1. Materials

Lignosulfonate (LS) was purchased from Jinzhou Ling Yu Chemical Co., Ltd. (Jinzhou, China). LS was obtained as the by-product of the sodium sulfite pulping process of pines. The purity, molecular weight and functional group information are shown in Table 1. Magnesium chloride (MgCl_2_), sodium carbonate anhydrous (Na_2_CO_3_) and potassium hydroxide (KOH) were obtained from Shanghai Macklin Biochemical Co., Ltd. (Shanghai, China). All chemical reagents used were analytical reagents (ARs) and used without further purification.

### 2.2. Preparation of Precursor

First, LS (0.50 g) was dissolved in 40 mL deionized water at room temperature and stirred for 0.5 h. Subsequently, MgCl_2_ (0.56 g) was added into the above solution, and the obtained mixed solution was named solution A after stirring for 2 h. Na_2_CO_3_ (0.63 g) was dissolved in 20 mL deionized water at room temperature, and the obtained mixed solution was named solution B after stirring for 0.5 h. Next, under continuous stirring, solution B was added dropwise to solution A, and additionally stirred for 2 h. The obtained precipitation was separated using centrifugation (8000 rpm) for 15 min and washed with deionized water. The LS/MgCO_3_ precursor was collected after vacuum drying (80 °C, 24 h).

LS (0.50 g) and KOH (0.50 g) were added into 30 mL deionized water at room temperature and stirred for 0.5 h. Then, the LS/KOH precursor was obtained after freeze-drying (−50 °C, vacuum pressure < 10 Pa, 24 h).

### 2.3. Preparation of LC, LPC and HLPC

The obtained precursor was transferred into a tube furnace for carbonization at 800 °C with a nitrogen atmosphere at the temperature increase rate of 10 °C/min. After carbonization, the sample was added into 0.1 M HCl solution and stirred for 3 h to remove the templates. The carbon materials were rinsed with distilled water and dried at 120 °C. Then samples were collected using a filter. The samples fabricated with LS, LS/KOH and LS/MgCO_3_ were named LC, LPC and HLPC, respectively.

### 2.4. Characterization

The morphologies of the samples were analyzed using the field emission scanning electron microscope (FE-SEM, ZEISS EVO 18 Research, CarlZeiss, Oberkochen, Germany) and the transmission electron microscope (TEM, JEOL 2200FS, JEOL Ltd., Tokyo, Japan). An X-ray diffraction (XRD) measurement was carried out with an X-ray diffractometer (D/MAX-1200, Rigaku Denki Co., Ltd., Tokyo, Japan) with Cu–Kα radiation, and scattering angles ranged from 10° to 80°. Raman spectra of samples were obtained using a Raman spectrometer (HORIBA Jobin Yvon Co., Ltd., Paris, France) using a 532 nm laser beam. X-ray photoelectron spectroscopy (XPS) was recorded with a Thermo Scientific (Waltham, MA, USA) K-Alpha X-ray photoelectron spectroscope. The N_2_ adsorption–desorption isotherms were obtained on a TriStar II 20 apparatus. The specific surface area was calculated with the Brunauer–Emmett–Teller (BET) method. The total pore volume and micropore volume were performed using the single-point method, and the mesopore volume was performed with the density function theory (DFT) method.

### 2.5. Electromagnetic Absorption Effectiveness

The carbonaceous material was added into paraffin with a fixed content of 7 wt%, and was then pressed into cylindrical-shaped specimens (*Φ*_out_ = 7.00 mm and *Φ*_in_ = 3.04 mm). The coaxial wire method was used to measure the complex permittivity and permeability values in the frequency range of 2–18 GHz with an Agilent N5230C PNA-L Network Analyzer (Santa Clara, CA, USA). The EMW absorption ability was evaluated through reflection loss (*RL*) values that were calculated according to the transmission line theory in the metal backboard model using Equations (1) and (2):

(1) $$Z_{\mathrm{in}}=Z_{0}\sqrt{\frac{\mu_{r}}{\varepsilon_{r}}}\tanh\left(\frac{2\pi jfd}{c}\sqrt{\mu_{r}\varepsilon_{r}}\right)$$

(2) $$\mathrm{RL}=20\log\left|\frac{Z_{\mathrm{in}}-Z_{0}}{Z_{\mathrm{in}}+Z_{0}}\right|$$

where *Z*_in_ and *Z*_0_ represent the input impedance and free space impedance; *ε*_r_, *µ*_r_ and *ε*_0_, *µ*_0_ are the complex permittivity and permeability of the absorber and free space, respectively; *f* is the frequency; *d* is the thickness of the absorber and *c* is the velocity of light in free space.

## 3. Results and Discussion

### 3.1. Synthesis and Characterization of HLPC

**Figure 1:**
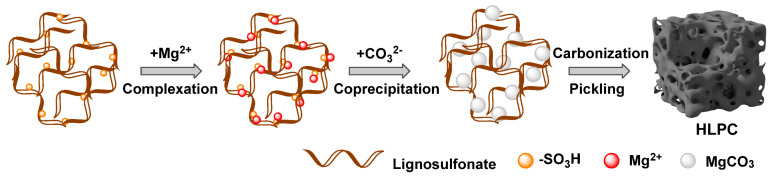
Schematic diagram of the fabrication process of HLPC.

The schematic illustration for brief formation processes of HLPC is shown in Figure 1, and LS and MgCO_3_ were used as the biomass carbon source and the template. The uniform mixing of the carbon source and the template is the key to enhance the porous structure of biomass-derived carbon [39]. First, Mg^2+^ was uniformly adsorbed onto LS in aqueous solution through complexation because of the abundant sulfonic acid group [40,41,42]. Then, MgCO_3_ precipitate was obtained in the mixed solution after the introduction of CO^3-^. Simultaneously, LS was deposited with MgCO_3_, and the uniformly mixed LS/MgCO_3_ precursor was obtained [43]. The thermal decomposition of MgCO_3_ to MgO and CO_2_ were occurred during carbonization. The generated CO_2_ causes a gas-phase exfoliation effect, which results in the formation of porous structures, especially macropores. In addition, the remaining MgO featured as a hard template, and the mesoporous structure was obtained after the removal of MgO through picking. The pyrolysis of oxygen-containing functional groups also created a microporous structure. Thus, biomass-derived carbon with a three-dimensional hierarchical porous structure was fabricated, and the porous structure was interconnected because of the uniform mixing of templates.

**Figure 2:**
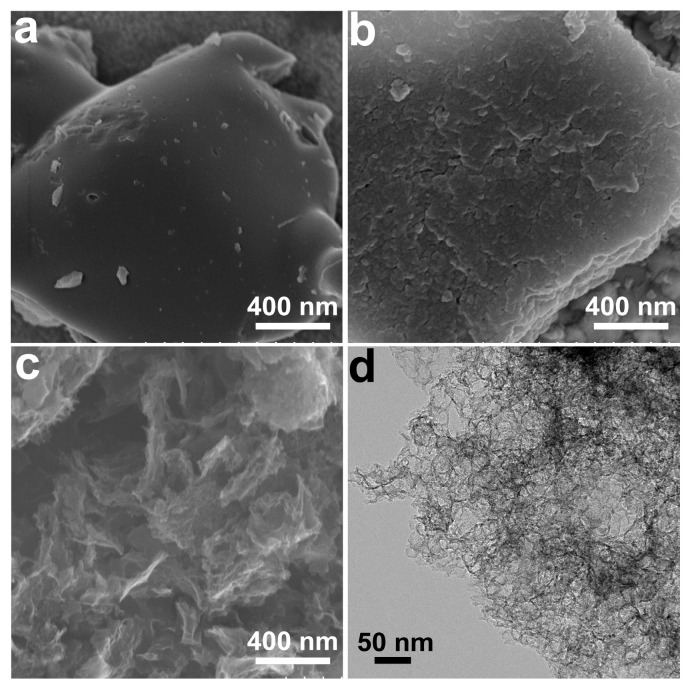
SEM images of (**a**) LC, (**b**) LPC and (**c**) HLPC. (**d**) TEM image of HLPC.

SEM and TEM were used to observe the morphologies of as-prepared carbonaceous materials. As shown in Figure 2a, there is no obvious pore structure in LC, and its surface is smooth. For LPC (Figure 2b), the surface is rough, and many narrow pores are observed. As shown in Figure 2c, HLPC presented a loose porous structure resulting from the removal of MgO after thermal decomposition of MgCO_3_. Interestingly, small-sized nanosheets were also observed from HLPC, which were due to the gas-phase exfoliation effect of CO_2_ from the thermal decomposition of MgCO_3_. In addition, the TEM image of HLPC (Figure 2d) indicates that the pores were connected. This unique construction of HLPC can greatly improve its pore volume.

**Figure 3:**
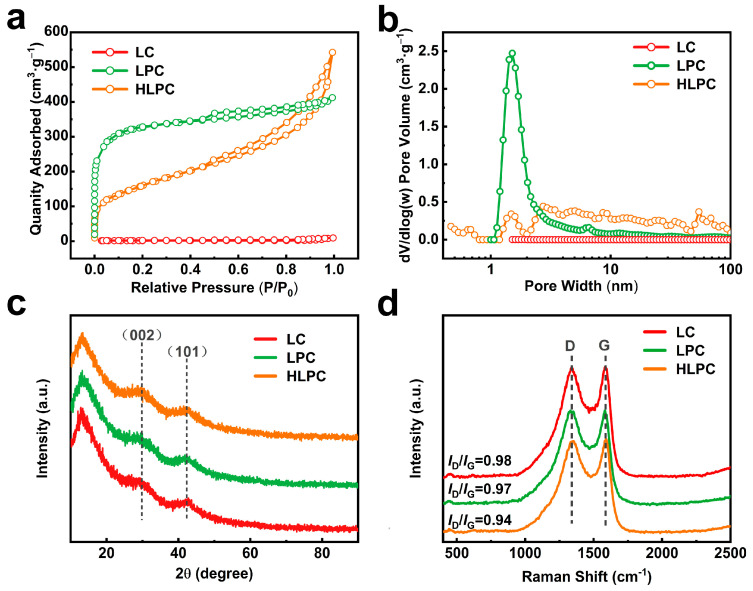
(**a**) N_2_ adsorption–desorption isotherms, (**b**) DFT pore size distribution, (**c**) XRD patterns and (**d**) Raman spectra of LC, LPC and HLPC.

The specific surface area and pore size distribution of obtained carbonaceous materials were further investigated using N_2_ adsorption–desorption isotherms, as shown in Figure 3a. The N_2_ adsorption–desorption capacity of LC was very low, indicating the poor porous structure. The isotherm of LPC was assigned to a type Ⅰ pattern with a narrow H4 hysteretic loop in the range of 0.5–1.0 P/P_0_, suggesting abundant micropores and slit pores. HLPC presented a combined type Ⅱ pattern with an H3 hysteretic loop in the range of 0.5–1.0 P/P_0_, implying the simultaneous presence of micropores, mesopores and macropores. DFT pore size distributions of LC, LPC and HLPC are shown in Figure 3b. LC showed little pore volume, while LPC had large amounts of micropores and small mesopores (2–8 nm). HLPC exhibited pore volume at the range of 0.5–100 nm, indicating the hierarchical porous structure.

The pore structure parameters and BET surface areas (*S*_BET_) of different samples are shown in Table 2. The *S*_BET_ of LC, LPC and HLPC were 5.1, 1224.4 and 586.5 m^2^/g, respectively. LPC presented the highest *S*_BET_, which benefited from the abundant micropores. The proportion of micropore volume in LPC reached 50.2%. HLPC, by contrast, presented the highest pore volume of 0.814 cm^3^/g, which was on account of abundant mesopores and macropores. 

XRD was used to analyze the crystal structures of LC, LPC and HLPC, as shown in Figure 3c. All these samples exhibited similar broad diffraction peaks around 28° and 42°, corresponding to (002) and (101) crystal planes of graphene, respectively. This result indicated that LC, LPC and HLPC were amorphous carbon.

The detailed characteristics of graphitization were evaluated using the Raman spectra, as exhibited in Figure 3d. Raman spectra of all samples presented two characteristic peaks at 1342 cm^−1^ (D peak) and 1578 cm^−1^ (G peak). The D peak corresponded to disordered sp^3^ hybridized carbon, while the G peak corresponded to sp^2^ hybridized carbon. Thus, the peak intensity ratio (*I*_D_/*I*_G_) was used to evaluate the graphitization level. The *I*_D_/*I*_G_ values for LC, LPC and HLPC were 0.98, 0.97 and 0.94, respectively. This indicated that HLPC presented a higher graphitization degree. It was possibly due to the gas-phase exfoliation effect of CO_2_ promoted the pyrolysis of LS.

**Figure 4:**
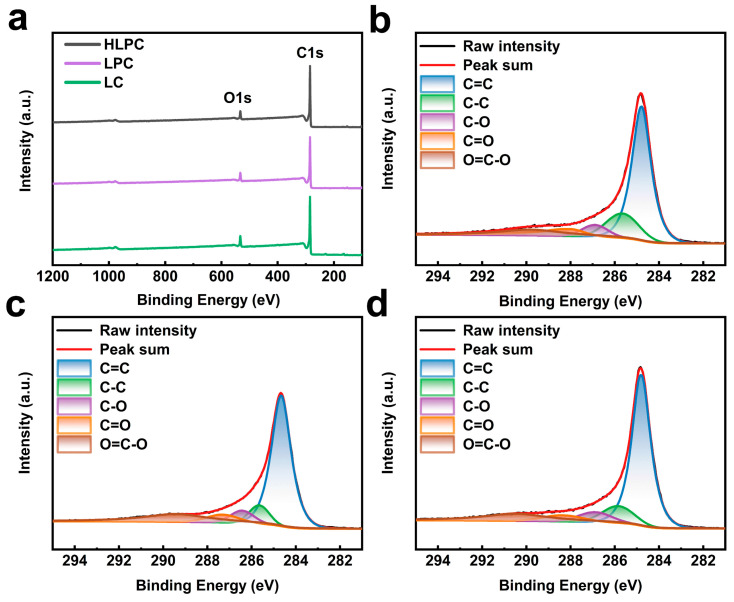
(**a**) XPS survey spectra of LC, LPC and HLPC. C1s spectra of (**b**) LC, (**c**) LPC and (**d**) HLPC.

X-ray photoelectron spectroscopy (XPS) was carried out for LC, LPC and HLPC to investigate the surface chemistry. As shown in Figure 4a, the XPS surveys indicated that the obtained carbonaceous materials were mainly composed of C and O elements. The proportion of elements are counted in Table S1, and HLPC presented the highest carbon content (92.28%), followed by LPC (91.15%) and LC (88.80%). As shown in Figure 4b–d, the C1s spectra of LC, LPC and HLPC can be deconvoluted into the following typical peaks of C species: C=C (284.8 eV), C-C (285.4 eV), C-O (286.5 eV), C=O (288.3 eV) and O=C-O (288.9 eV). In addition, the ratio of peak integral area was used to calculate the relative content of C species, and the result is shown in Table S2. HLPC presented the most C=C content, which was consistent with the Raman spectra.

### 3.2. EMW Absorption Performance of LC, LPC and HLPC

**Figure 5:**
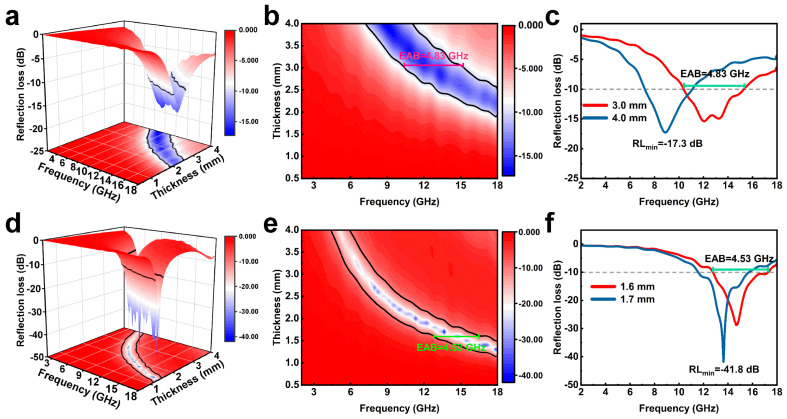
Three-dimensional reflection loss representations, two-dimensional reflection loss projection mappings and reflection loss curves of (**a**–**c**) LPC and (**d**–**f**) HLPC.

To investigate the EMW absorption performance of samples, the *RL* values were calculated based on the transmission line theory with Equations (1) and (2). When the *RL* value was lower than −10 dB, almost 90% of microwaves can be attenuated, which is also considered the threshold for effective EMW absorption. Three-dimensional *RL* values versus frequency for LC, LPC and HLPC are shown in Figure S1a and Figure 5a,d. For LC, the *RL* values were greater than −10 dB over the full frequency band in the thickness range of 0.5~4.0 mm, indicating the poor EMW absorption performance. In comparison, the *RL* values of LPC and HLPC can fall below −10 dB at a certain frequency range through controlling the thickness. From the two-dimensional colored *RL* values of LPC and HLPC (Figure 5b,e), the absorption peak shifts to a lower frequency region with an increase in the thickness, which was consistent with carbon-based materials reported in the literature [23]. And this can also be described using the 1/4 wavelength cancelation law.

LPC exhibited the minimum *RL* (*RL*_min_) value of −17.3 dB (8.9 GHz, 4.0 mm), and the optimal effective absorption bandwidth (EAB, *RL*_min_ ≤ −10 dB) is 4.83 GHz at 3.0 mm. For HLPC, the *RL*_min_ value of −41.8 dB was achieved at 13.6 GHz (1.7 mm), while the optimal EAB is 4.53 GHz with a thickness of 1.6 mm. The *RL* curves of LPC and HLPC are shown in Figure 5c,f with corresponding thickness. In an ultralow filler ratio of 7 wt%, HLPC presented the lower *RL*_min_ value, and the matching thickness was thinner. In addition, the EAB values were similar for LPC and HLPC, but the matching thickness of HLPC was thinner.

**Figure 6:**
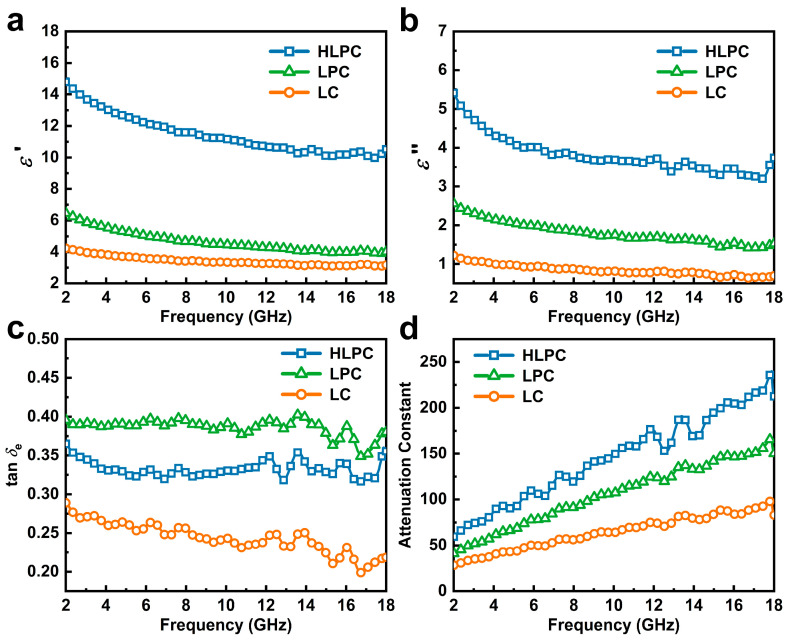
(**a**) Real part of permittivity, (**b**) imaginary part of permittivity, (**c**) dielectric loss tangent and (**d**) attenuation constant *α* for LC, LPC and HLPC.

To explain the mechanisms of EMW absorption, the permittivity and permeability were analyzed further. The real parts (*μ*′) and imaginary parts (*μ*″) of permeability are shown in Figure S2. For all carbonaceous materials, *μ*′ and *μ*″ kept 1.2 and 0 with the whole frequency range, indicating a nonmagnetic nature. Samples presented a weak magnetic loss capability, and permeability was not further analyzed in detail. The real parts (*ε*′) and imaginary parts (*ε*″) of permittivity are shown in Figure 6a,b. The *ε*′ values of LC, LPC and HLPC exhibited a similar declining trend over 2–18 GHz, owing to the frequency dispersion. For HLPC, the *ε*′ value decreased from 14.9 to 10.6 with the increase in frequency from 2 GHz to 18 GHz, which was higher than that of LPC (6.9 to 3.9) and LC (4.3 to 3.1). The higher *ε*′ values implied better conductivity, which is unfavorable for EMWs entering the absorber. In other words, a small *ε*′ value was demonstrated to be more effective for EMW energy storage, which was called an impedance match. In the meantime, the *ε*″ values declined from 5.4 to 3.6 for HLPC, from 2.6 to 1.5 for LPC and from 1.2 to 0.7 for LC. The higher *ε*″ values indicated better dielectric attenuation capabilities for HLPC, which were mainly attributed to the high graphitization degrees and abundant hierarchical porous structure.

The dielectric loss ability of carbonaceous materials was jointly determined using *ε*′ and *ε*″ values. Dielectric loss tangent (tan *δ*_e_ = *ε*″/*ε*′) can be used to evaluate the dielectric loss capacity of the absorber and the tan *δ*_e_ values of LC, LPC and HLPC are shown in Figure 6c. LPC and HLPC showed a higher tan *δ*_e_ value, indicating the better dielectric loss capacity. Compared with LC, LPC and HLPC exhibited a developed porous structure, which provides an abundant air/carbon heterogeneous interface. Thus, the interfacial polarization was enhanced. Significantly, the tan *δ*_e_ value of LPC was slightly more than that of HLPC. The XPS result showed more oxygen in LPC, and that the oxygen-containing functional groups could produce more dipole polarization.

Additionally, the attenuation constant (α) was calculated to assess the dissipation ability of EMWs inside the carbonaceous materials with the following Equation (3):

(3) $$\alpha =\frac{\sqrt{2}\pi f}{c}\times \sqrt{\left(\mu^{\prime \prime }\varepsilon^{\prime \prime }-\mu^{\prime }\varepsilon^{\prime }\right)+\sqrt{{\left(\mu^{\prime \prime }\varepsilon^{\prime \prime }-\mu^{\prime }\varepsilon^{\prime }\right)}^{2}+{\left(\mu^{\prime }\varepsilon^{\prime \prime }-\mu^{\prime \prime }\varepsilon^{\prime }\right)}^{2}}}$$

As shown in Figure 6d, the α values of LC, LPC and HLPC presented an increasing trend with the increase in frequency. The α value of HLPC was the maximum, suggesting the strongest dissipation ability for EMWs. This was attributed to the unique three-dimensional hierarchical porous structure of HLPC, which provided an abundant interconnected conduction network and enhanced the conduction losses.

**Figure 7:**
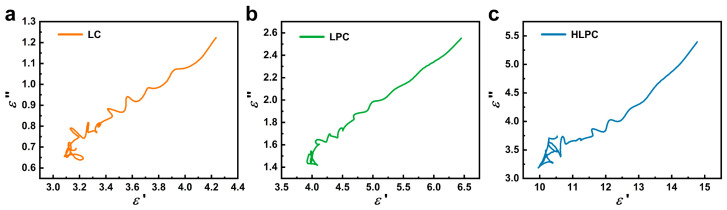
Cole–Cole semicircles (ε′ versus ε″) of (**a**) LC, (**b**) LPC and (**c**) HLPC.

Cole–Cole plots of samples based on the Debye theory are shown in Figure 7. In the Cole–Cole plots of LC, LPC and HLPC, several semicircles were observed, which demonstrated the existence of multiple polarization relaxation processes after the entering of EMWs. In addition, the plots all present a straight tail, representing conduction loss, and the slope of the straight tail is positively correlated with conductivity, so HLPC has the best conductivity. This result was consistent with Raman and XPS analyses.

### 3.3. EMW Absorption Mechanism

**Figure 8:**
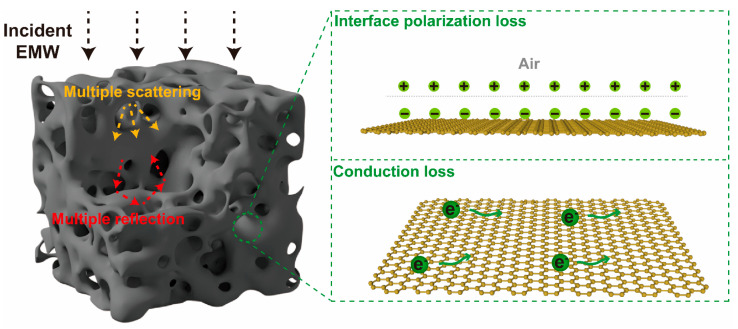
Schematic diagram of EMW absorption mechanisms for HLPC.

Based on the above results, the EMW absorption mechanisms of HLPC are delineated in Figure 8. The incident EMW reached the surface of HLPC, and the reflection and scattering occurred because of the 3D hierarchical porous structure. Therefore, the transmission paths of EMWs were prolonged, which was beneficial to the dissipation process. For HLPC, the loss of EMWs mainly originated from two sources. On the one hand, HLPC presented a large pore volume, providing an abundant gas–solid interface between the air and the carbon for interfacial polarization. On the other hand, HLPC presented a unique 3D hierarchical porous structure compared with LC and LPC, which endowed its interconnected carbon skeleton. The conductive network was constructed, enriching the efficient paths for the migration of electrons. Thus, the conduction loss was enhanced. Overall, the large pore volume and 3D hierarchical porous structure enabled HLPC to present an excellent EMW absorption ability.

## 4. Conclusions

In conclusion, a carbonaceous material with 3D hierarchical porous structures was successfully synthesized with LS as the carbon precursor through an adsorption and carbonization process. By comparison with LPC and LC, HLPC presented excellent EMW absorption performance. For HLPC, the *RL*_min_ value of −41.8 dB and optimal EAB of 4.53 GHz were achieved with the matching thickness of 1.7 mm and 1.6 mm. In addition, this excellent performance was obtained at a low filling ratio of 7 wt%. The favorable EMW absorption performance resulted from the high pore volume and 3D hierarchically porous carbon network of HLPC. This work provides a new strategy to prepare lignin-derived carbon with excellent EMW absorption performance.

## Figures and Tables

**Table 1:** Purity, molecular weight and functional group information of LS.

Sample	Purity (%)	M_w_ (g/mol)	Phenolic OH (mmol/g)	Carboxy (mmol/g)	Sulfonic Group (mmol/g)
LS	95.1	19,400	1.87	1.52	1.57

**Table 2:** Specific surface area and pore volume parameters of LC, LPC and HLPC.

Sample	*S*_BET_ (m^2^/g)	*V*_total_ (cm^3^/g)	*V*_micro_ (cm^3^/g)	*C*_micro_ (%)	*V*_meso_ (cm^3^/g)	*C*_meso_ (%)
LC	5.1	0.0140	0.0007	4.9	0.0040	31.6
LPC	1224.4	0.6362	0.3200	50.2	0.1751	28.9
HLPC	586.5	0.8145	0.0064	0.8	0.4006	71.1

## Data Availability

Further relevant data can be provided on request.

## Supplementary Materials

The following supporting information can be downloaded at: https://www.mdpi.com/article/10.3390/polym16020201/s1, Figure S1: Three-dimensional reflection loss representations and two-dimensional reflection loss projection mappings of LC; Figure S2: (a) Real part and (b) imaginary part of permeability for LC, LPC and HLPC; Table S1: Elements content of samples determined by XPS; Table S2: Functional groups content of samples determined by XPS; Table S3: Functional groups content of samples determined by XPS.
