# Supplementary material for: Production of Lignin-Derived Functional Material for Efficient Electromagnetic Wave Absorption with an Ultralow Filler Ratio
Source: Polymers (Basel). 2024 Jan 10;16(2):201. doi: 10.3390/polym16020201 (PMC10819316; doi:10.3390/polym16020201)
Supplement: Supplementary file 1 [file polymers-16-00201-s001.zip › polymers-2784932-supplementary.pdf]

Production of Lignin-Derived Functional Material for Efficient Electromagnetic Wave Absorption with an Ultralow Filler Ratio

Yuebin Xi [1], Xingxiang Ji [1], Fangong Kong [1], Tianjin Li [1,2,*] and Binpeng Zhang [3,*]

[1] Key Laboratory of Pulp and Paper Science and Technology of Ministry of Education, State Key Laboratory of Biobased Material and Green Papermaking, Qilu University of Technology (Shandong Academy of Sciences), Jinan 250353, China; qlgdxyb@qlu.edu.cn (Y.X.); jxx@qlu.edu.cn (X.J.); kfg@qlu.edu.cn (F.K.)

[2] Energy Institute, Qilu University of Technology (Shandong Academy of Sciences), Jinan 250353, China

[3] School of Chemistry and Chemical Engineering, Henan University of Technology, Zhengzhou 450001, China

* Correspondence: litj@qlu.edu.cn (T.L.); bpzhang@haut.edu.cn (B.Z.)

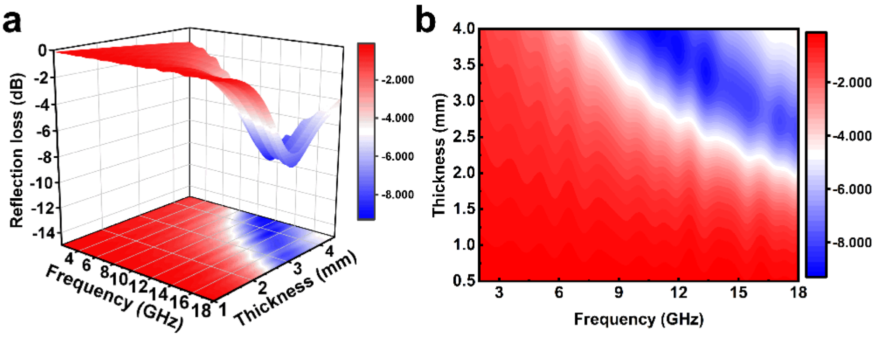

Figure S1. Three-dimensional reflection loss representations and two-dimensional reflection loss projection mappings of LC.

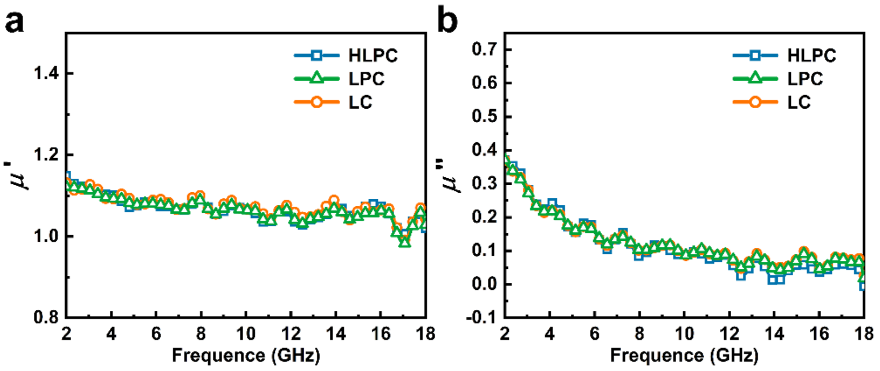

Figure S2. (a) Real part and (b) imaginary part of permeability for LC, LPC and HLPC.

Table S1 Purity, molecular weight, and functional group information of LS

Sample	Purity (%)	M_w (g/mol)	Phenolic OH (mmol/g)	Carboxy (mmol/g)	Sulfonic group (mmol/g)
LS	95.1	19400	1.87	1.52	1.57

Table S2 Elements content of samples determined by XPS.

Sample	Element content (%)			
	C	O	N	S
LC	88.80	8.57	1.07	1.56
LPC	91.15	7.78	0.73	0.34
HLPC	92.28	6.78	0.57	0.37

Table S3 Functional groups content of samples determined by XPS.

Sample	C 1s content (%)				
	C=C	C-C	C-O	C=O	COOH
LC	59.11	16.33	6.17	8.03	10.36
LPC	64.68	8.31	6.43	5.66	14.92
HLPC	64.90	10.84	6.60	5.00	12.66
